# Assessment of the Propulsion System Operation of the Ships Equipped with the Air Lubrication System

**DOI:** 10.3390/s21041357

**Published:** 2021-02-14

**Authors:** Mariusz Giernalczyk, Piotr Kaminski

**Affiliations:** Faculty of Marine Engineering, Gdynia Maritime University, 81-234 Gdynia, Poland; p.kaminski@wm.umg.edu.pl

**Keywords:** emission reduction, air lubrication system, drag reduction, energy efficiency design index

## Abstract

This paper is an attempt to evaluate the effectiveness of the ship’s hull air lubrication system in order to reduce the drag leading to fuel consumption reduction by ships. The available papers and reports were analyzed, in which records of the operation parameters of the propulsion system of ships equipped with this system were presented. These reports clearly show the advantages of using air lubrication system. On the basis of collected operating parameters of the propulsion system the authors performed analysis of operation effectiveness of the Air Lubrication System on the modern passenger ship was. The results of this analysis do not allow for a clearly positive opinion about its effectiveness. Additionally, the conditions that should be met for the system to be more effective and to significantly increase the propulsion efficiency were indicated.

## 1. Introduction

The MARPOL Annex VI came into force on 19 May 2005, concerns on the prevention of air pollution by ships. It forced the ship-owners to apply solutions aimed at reducing the emission of harmful substances, such as nitrogen oxides (NOx), sulphur oxides (SOx), carbon oxides (CO), hydrocarbons (HC) and particulate matter (PM) into the atmosphere. This annex did not initially include carbon dioxide emission reductions. However, international institutions including the International Maritime Organization (IMO) have noticed the threat of the greenhouse effect, caused in a large scale by carbon dioxide. In July 2011, the Annex VI of the MARPOL Convention was extended by Chapter IV that aims to reduce greenhouse gases emissions in particular carbon dioxide by ships [1,2].

**Figure 1:**
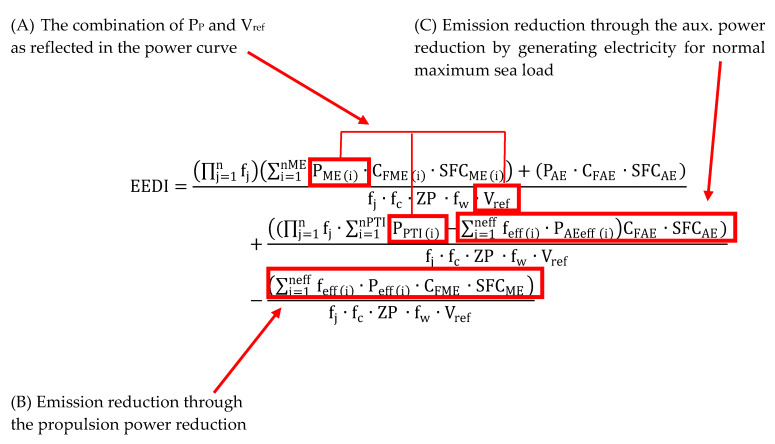
The Energy Efficiency Design Index (EEDI) formula with indicated elements that may affect the emission reduction by the use of ALS.

The reduction of CO_2_ emissions is to be achieved by introducing for all newly built vessels greater than 400BRT, the Energy Efficiency Design Index (EEDI) [3]. The EEDI index is defined as the ratio of the amount of CO_2_ [g] to the amount of cargo [t] on a specific shipping distance [Mm] and is a specific balance between the social benefit of cargo transport and the negative phenomenon of CO_2_ emissions to the atmosphere. It is to be used as a tool to indirect control of CO_2_ emissions and to increase the energy efficiency of ships power plants. The EEDI value for the ship is calculated in accordance to the procedure contained in Resolution MEPC.308(73) [4] and must be equal to or lower than the value required for the type and size of the vessel. It is calculated based on the formula presented on Figure 1.

In 2018, the IMO published a preliminary strategy to reduction of the greenhouse gases emissions reduction from ships with the principles of its application [4]. This forced ship-owners to search technological solutions aimed at reducing carbon dioxide emissions and improving sailing efficiency by decreasing fuel consumption. These goals can be achieved, inter alia, by reducing the vessel’s hydrodynamic resistance [5,6,7,8,9]. One of the methods to reducing the drag by reducing frictional resistance is insertion of an air layer between the underwater part of ship’s hull and water. The air bubbles in this method are used as lubricant and it is called Air Lubrication (AL) [10,11,12]. AL systems (ALS) are recognized by IMO as category B-1 (Innovative Energy Efficiency Technology) as described in MEPC.1/Circ.815 [4]. This technology significantly lowers the EEDI value, mainly by reducing the components surrounded by the frame in formula (Figure 1).

## 2. Ship Hull Resistance

The ship moves on the boundary of two fluids-air and water, which counteract movement by causing hydrodynamic and aerodynamic forces that, create movement drag. The total resistance R includes the sum of the aerodynamic resistance R_A_ and the hydrodynamic resistance R_H_. The hydrodynamic resistance R_H_ is the sum of the components of the frictional resistance R_F_ and the pressure R_P_ (wave resistance R_W_ and viscous pressure resistance R_VP_). Thus, the total resistance of a ship moving through the water is given by the formula:

R = R_F_ + R_W_ + R_VP_ +R_A_ (1)

**Figure 2:**
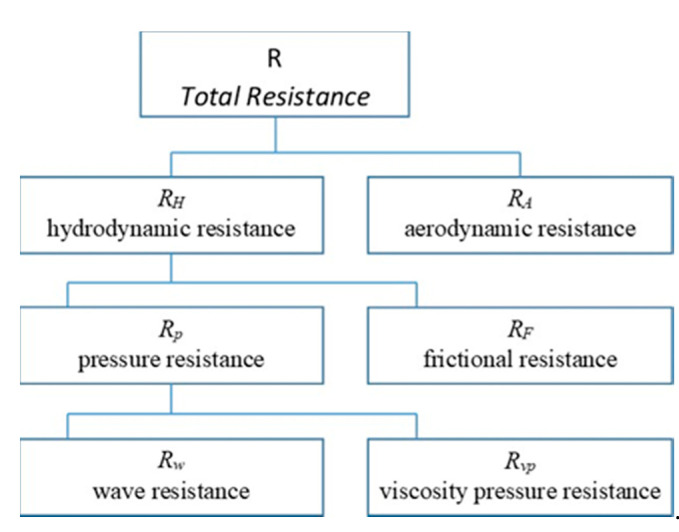
The total hull resistance structure [13].

The structure of the total hull resistance is shown on Figure 2.

**Figure 3:**
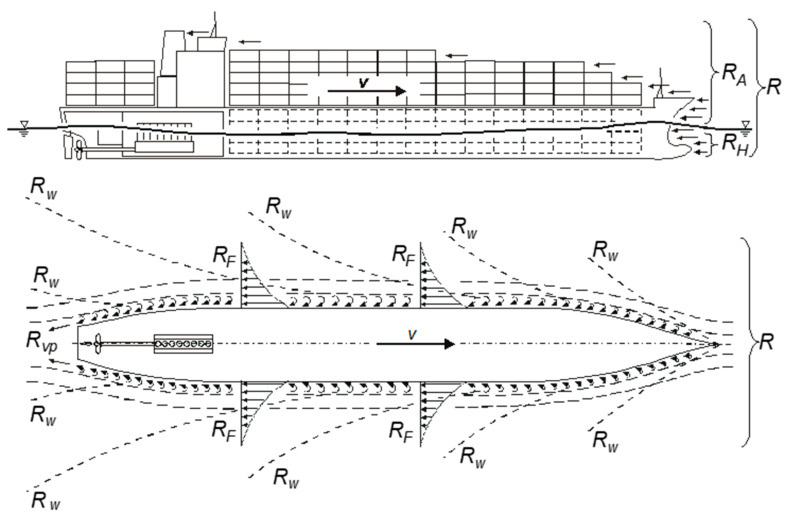
Distribution of the total hull resistance [13].

The distribution of the total resistance components is presented in Figure 3. Both, hydrodynamic and aerodynamic resistance are described by the general resistance forces equation:

(2) $$R=c\cdot \frac{\rho \cdot v^{2}}{2}\cdot S$$

where, *R* is the drag force, *c* the dimensionless drag coefficient, *ρ* is the fluid density, *v* is the velocity, *S* is the hull surface in fluid.

The individual components of a ship total resistance affect its size to a different extent [14,15]. The pressure R_P_ (viscous pressure resistance R_VP_ and in particular, wave resistance R_W_) and frictional resistance have the greatest share in the structure of total resistance. The shape of the hull-its slenderness, fullness and the speed of the vessel significantly influence the wave resistance R_W_. For lower sailing speeds, the average value of the wave resistance is 8 ÷ 25% of the total resistance while at high sailing speeds it may reach the value of 40 ÷ 65% of the total resistance [13]. In order to minimize the resistance associated with sea waves the hull shape is optimized already at the design stage. Designers use computer simulations during design and then models’ tests in the ship model basin in order to reduce this resistance to a minimum.

On other hand, the frictional resistance increases with the ship’s operation (service) time. It is caused by an increase of roughness of the underwater part of the hull as a result of its overgrowing with seaweed, crustaceans, algae, mollusks and other organisms living in the water. It is estimated that from the moment the ship leaves the dry dock, the daily increase of resistance due to fouling of the hull is 0.2 ÷ 0.5% of the total resistance, although there are lower values for colder waters and higher values for warmer waters riche in flora and fauna [16,17,18,19].

To reduce viscosity friction the area of the hull wetted surface needs to be reduced. This can be performed by separating the underwater part of the hull surface from the water with a layer of air [20]. The general term used to describe this phenomenon is called hull “Air Lubrication” (AL).

The remaining components have a smaller influence on the total resistance, although it they may be different in particular ship sailing conditions. A good example can be large container ships, where during the ship’s movement in a direction opposite to a very strong wind the containers loaded on board (even up to ten layers) create above water part of the hull resistance (aerodynamic resistance) which is a significant share in the total resistance of the ship. 

## 3. Method to Reduce the Ship’s Hull Resistance by Introducing an Air Layer under the Hull

**Figure 4:**
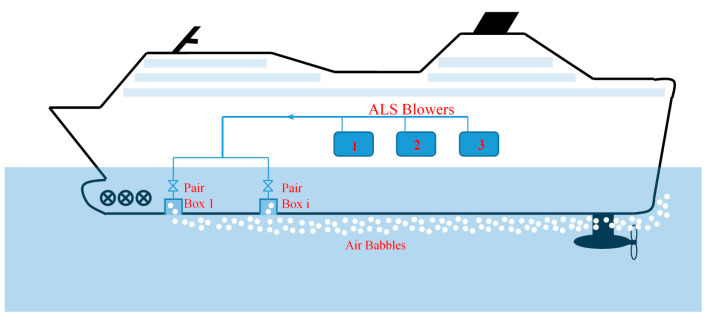
Diagram of the system of introducing the air layer under the hull bottom of the passenger vessel-Air Lubrication System (ALS).

Surface frictional resistance is proportional to the wetted surface of the ship’s hull; therefore the ALS works on a simple principle of keeping a layer of air bubbles under the hull [21]. The method of producing and introducing the air layer under the bottom of the passenger ship’s hull is presented in Figure 4 [22,23,24].

**Figure 5:**
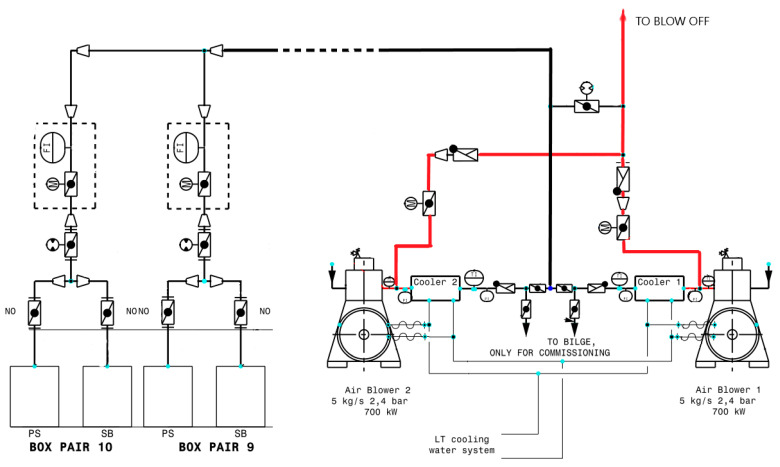
Compressed air supply system (ALS) of the underwater part of the hull on a large cruise liner.

High-capacity blowers are used to generate air bubbles that flow at a constant speed under bottom of the hull. The air bubble outlets are located along the bottom of the hull, symmetrically on both sides of the ship’s center line [13,23]. A schematic diagram of the ALS with two blowers, distribution line of compressed air and with air distribution boxes on a large passenger vessel (cruise liner) is presented in Figure 5.

The blowers forced compressed air to 20 distribution boxes (10 pairs) which are a structural element of the ship’s hull. Two distribution boxes: one box on the port side and one on the port side are symmetrically connected to one supply subline. The boxes are equipped with corrosion protection (zinc anodes). The compressors run at a constant speed and are controlled by a control system, which can reduce the capacity of one compressor to about 45% of nominal value. This is executed by regulating the air supply with steering wheel with variable angle blades. In this way the energy consumption of the blowers driving motors can be reduced.

The ALS method may be applied at the design stage and built on a new vessel as well as installed on the vessel after a certain period of operation. The introduction of ALS on operated ship is a complicated process and requires comprehensive analyses, calculations, measurements and most often computer simulations [24]. There are several companies specializing in the design and installation of ALS on the vessels and each company calls this system otherwise i.e.: Mitsubishi Co. – Mitsubishi Air Lubrication System (MALS), R&D Engineering – Winged Air Induction Pipe System (WAIP), Samsung Heavy Industries – SAVER System (SAVER Air), Silverstream-Silverstream System, Foreship-Foreship Air Lubrication System (Foreship ALS) and others [5]. The first installation of the ALS called Silverstream System (Addlestone, UK) was applied on a chemical tanker MT Amalienborg with a carrying capacity of 40,000 DWT. This vessel was equipped for propulsion with a low-speed B&W 6S50MC main engine with power of 13,452 BHP [25]. After installing this system on the ship many operation parameters when ALS was ON and OFF were recorded, among others: propulsion system operation parameters and ship speed (on water and GPS), as well as power consumption by blowers, main engine speed, shaft power (torque), fuel consumption by main engines. And additionally, weather conditions (hydrometeorological conditions).

**Figure 6:**
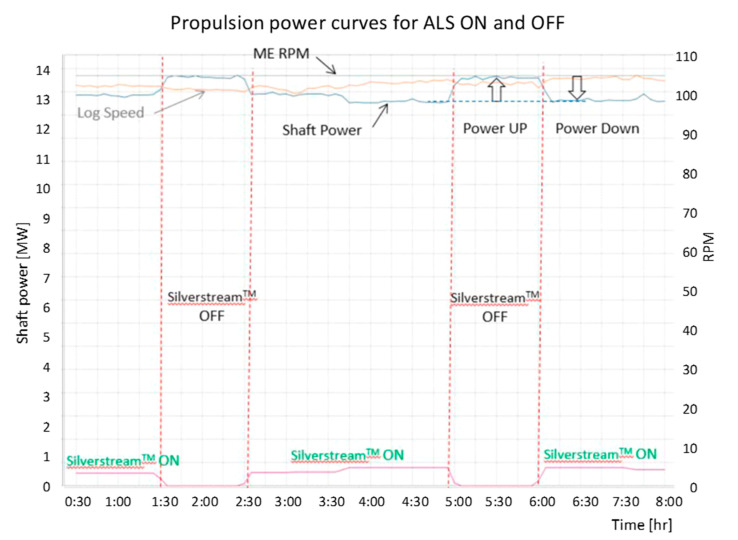
Example of Air Lubrication Effect from Monitoring System on MT Amalienborg [26].

Figure 6 shows the impact of ALS (Silverstream system) operation installed on MT Amalienborg on the changes of the propulsion power (shaft power) and ship speed. The course of the parameters presented in this diagram shows that, while maintaining a constant rpm of main engine, activation of the ALS system causes a decrease in the propulsion power demand and does not significantly affect ship speed, moreover the lower part of the diagram shows the energy consumption by ALS blowers.

**Figure 7:**
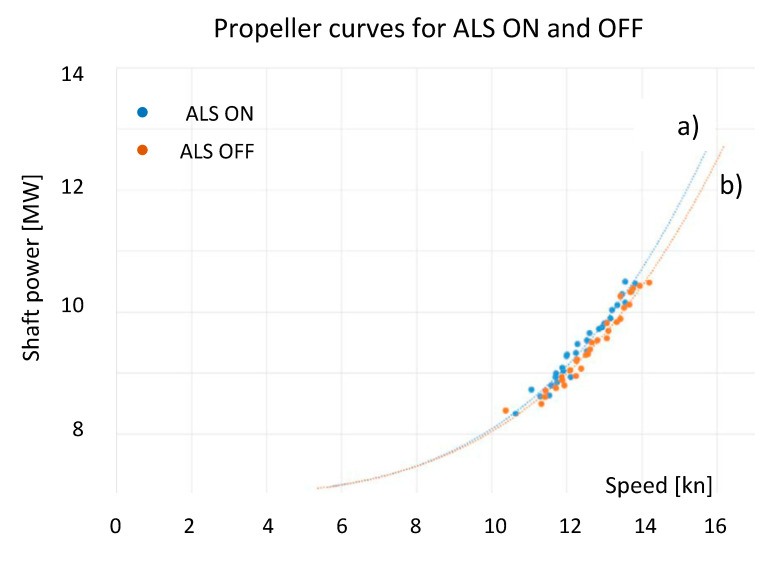
Average Shaft Power for given RPM (Revolution Per Minute) against Speed from MT Amalienborg [26].

Using the operating parameters of the propulsion system during the sea test of the ALS system the power consumption curve as a function of ship speed (propeller curve) were prepared, shown in Figure 7.

The propeller curve for ON and OFF ALS system presented in the diagram P = f (v) shows the benefits of this system operation in the form of lower demand for propulsion power at the same ship speed. This is evidenced by the shift of the propeller curve (a) towards the so-called lighter propeller curve (b).

Measurements results taken on a vessel with ALS were processed by the authors [26,27] and allowed to determine the net energy savings required to propel the vessel, amounting to about: 0.1 ÷ 4.5% (at the sailing speed of 11 ÷ 14 kn). Although these results took into account additional losses related to the blowers drive energy and the resistance of the air distribution boxes, the method of data processing and the obtained results is not precisely explained in the study.

## 4. Assessment of the ALS Operation

Promising results of operation obtained after installing the ALS on the chemical tanker Amalienborg and other ships encouraged many ship-owners to install this system on their vessels, especially on cruise ships [28,29,30]. The Silverstream systems were installed, among others on Carnival cruisers (Sapphire Princess, Diamond Princess). On the other hand, the Foreship ALS were installed among others on Royal Caribbean International’s large, modern passenger ships. The list of cruise ships equipped with the ALS and delivered up to 2019 is shown in Table 1 [5].

The ALS system has been installed on one of the large cruise liners since the ship was put into service. To generate air with the required parameters, two single-stage centrifugal blowers integrated with the gearbox, driven by a motor with power of 700 kW each and a capacity of 5 kg/s at an overpressure of 1.4 bar, were installed on the ship.

**Figure 8:**
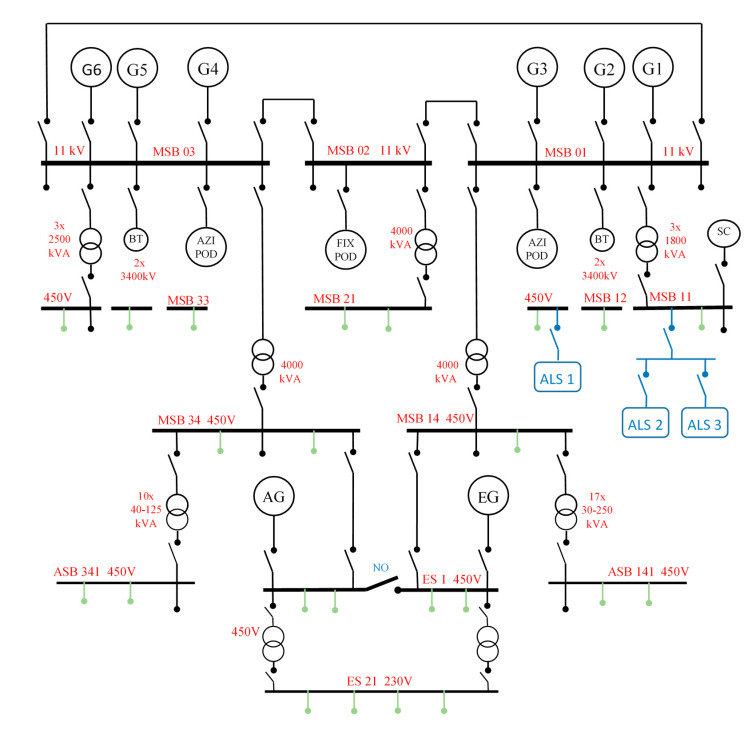
Ae example of simplified diagram of power distribution on a passenger ship with diesel-electric propulsion system.

On similar passenger vessels, the ALS was installed after some of operation time. Ship-owner decided to install three blowers similar to the ones on the previous cruiser. These vessels are equipped with diesel-electric propulsion system, consisting of six engines driving the main generators with the capacity of 12600 kW each, and two auxiliary generators with a capacity of 2500 kW each. Distribution of the generated electric power is shown in Figure 8.

Electricity is generated by main generators (G1÷G6) and auxiliary generators (AG+EG). The highest voltage current (11 kV) is directed through the Main Bus Bars (MBB) to supply three gondola propellers (2 × AZIPOD + 1 × FIXPOD) and four bow thrusters (BT). Main Switchboard Bus bars (MSB) with a voltage of 450 V power most of the machines and devices in the engine room, including ALS blowers, while the receivers with the lowest power are supplied with 230 V.

After starting the vessel operation with ALS installed, modified propulsion system was tested. Operating parameters of the propulsion system were recorded with the ALS ON and OFF. The system was turned on for a period of 2 ÷ 3 h, and the parameters were recorded before turning on, during operation and after turning off the system. Due to the relatively short time intervals (30 ÷ 60 min) between the recording of parameters it was assumed that the weather conditions were constant. Table 1 shows the recorded and calculated parameters such as energy consumption for propulsion, energy consumption for the other needs of the vessel, the power used by the ALS blowers, fuel consumption of generators engines, etc.

**Figure 9:**
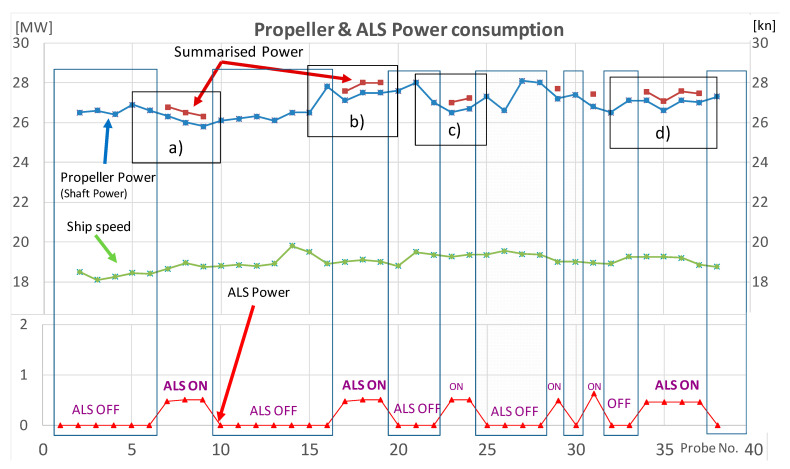
Air Lubrication Effect from Monitoring System on the large cruise liner.

Based on the data from Table 1, the variability of selected parameters is shown in Figure 9. It presented the impact of the ALS operation on the change of the propeller power PP (Propeller Power/Shaft Power), vessel speed and the power used by blowers ALSP (Air Lubrication System Power). It also took into account the summary power SPP (Summary Propulsion Power) used for the ship propulsion and to drive ALS blowers. The list of selected i.e., measured and calculated parameters is presented in Table 2.

**Figure 10:**
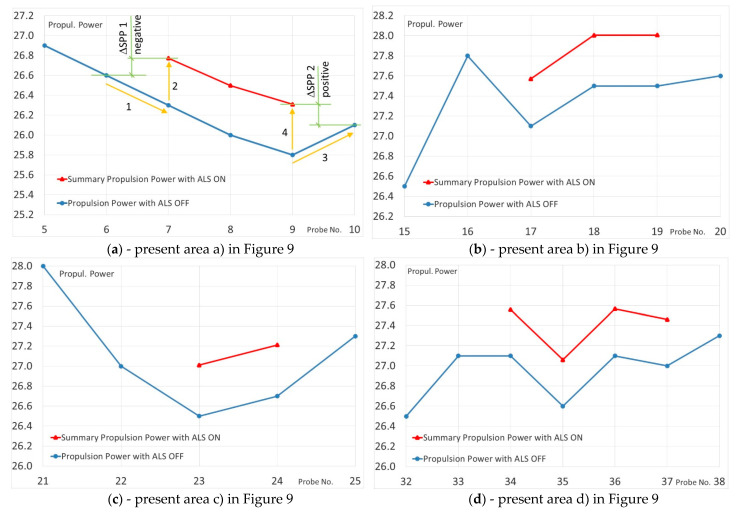
Details of changes in consumption of Propulsion Power and Summary Propulsion Power with ALS (Air Lubrication System) switched ON and ALS switched OFF.

The course (track) of parameter variability shown in the diagram (Figure 8) is general and does not allow for a detailed analysis of the system operation in particular periods, i.e., with ALS on and off. Therefore, on the Figure 10 are presented fragments of the diagram where the ALS was started and stopped in detailed.

As shown in Figure 10a after switching ON the ALS at measuring point (probe 7) if compared to point 6 there is a decrease (vector 1) of propeller power (PP) consumption (AZIPOD’S + FIXPOD) but at the same time there appeared a demand for energy to drive blowers ALSP (vector 2). Comparing summary power of SPP before and after starting the ALS, a slight increase of the value ΔSPP1 (+0.3 MW) is observed, with a minimal increase of ship’s speed (0.3 kn) (the line of the ship’s speed in Figure 9). Between the measurement points 9 (probe 9) and 10 (Figure 10a) as a result of the ALS OFF there is a change in the SPP power distribution, due to the lack of power demand for the ALS blowers drive ALSP (line 3) and increase of propeller power (PP) (line 4) with the simultaneous lack of vessel speed changes. This causes only a slight decrease of demand for the summary power ΔSPP2 (0.3 MW).

Similar changes in the distribution of the summary power SPP can be observed when switching the ALS ON and OFF shown on Figure 10b–d.

In Figure 10b is observed a decrease of the summary power SPP is observed demand when ALS is turned ON (transition from point 16 to 17), at constant ship speed. However, after switching the ALS OFF (transition from point 19 to 20) there is a slight increase in propeller power PP and a simultaneous minimal decrease in ship speed.

Figure 10c presents the operating parameters of the propulsion system before (point 22) and after switching the ALS ON (point 23). Switching the ALS into operation does not increase the summary power SPP for propulsion (the power transmitted to the PP ship propulsion decreases, but the ALSP blower propulsion power appears with the same value, at a minimal decrease in ship speed (0.1 kn).

In Figure 10d it is observed that after the ALS is turned ON into operation (points 33 to 34) the propeller power PP demand does not change but the summary power SPP for propulsion increases by the ALSP value (ALS blowers drive power). At the next measurement, points (34÷36) there are fluctuations in the summary power SPP demand without changing of ALSP at the same speed of the ship. When the ALS is turned OFF (transition from point 37 to 38) the propeller power PP demand increases but it is less than the power consumed by the blowers ALSP. At the same time, the speed of the ship drops slightly by about 0.2 kn. 

Only at measurement points, 17 and 29 (Figure 9) there are visible slight benefits of switching the ALS ON are visible in the form of decrease the summary propulsion power SPP at unchanged ship speed. Only these points confirm the assumption that ALS reduces fuel consumption for the ship propulsion. Other data do not confirm this assumption.

Moreover, when observing the fuel consumption (Table 1, column D) it can be noticed that switching ALS ON causes an increase or decrease of fuel consumption by approx. −0.8% (positive effect of ALS activation) to 1.7% (negative effect) respectively. In addition, when switching ALS OFF an increase or decrease approx. −0.9% to 2.4 can be observed respectively. It is accompanied by a minimal change in the speed of the ship. 

**Figure 11:**
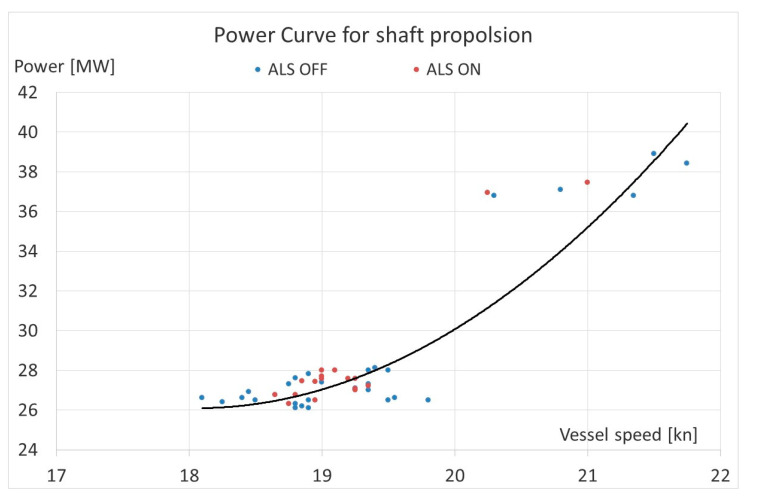
Average Shaft Power for given RPM against Speed from on cruise liner with diesel-electric propulsion.

Additionally, based on the collected data, the propulsion power in the ship speed curve (propeller characteristic) was drawn. It was done for propulsion with ALS ON and OFF and presented by points and trend lines in Figure 11. This diagram also confirms that the ALS operation does not increase efficiency of propulsion system. This is due to the location of the points, especially the lack of clear separation between operating points for ALS ON and OFF (like for the MT Amalienborg in Figure 7).

## 5. Discussion

This paper presents a more critical assessment of the operation of the ALS system than in the presented and available reports and publications. It should be noted that the presented analyses of the ALS (Silverstream) installed on MT Amalienborg show that the benefits (savings) resulting from its use are about 4.5% at the vessel’s speed of 14 kn. However, the attention should be paid to the fact that the benefits of using this system at speeds below 14 kn are doubtful. The net savings dropped to the amount of 3.2% at 13 kn, 1.7% at 12 kn. Taking into account the fact that the vessel does not always sail at the maximum design speed (14.5 kn for MT Amalienborg) and take into considering the investment and service costs the benefits of using this system seem to be questionable.

On the other hand, on the basis of the observations, analyses and records of operating parameters for the passenger vessel with diesel-electric propulsion system, it can be concluded that the activation of the ALS resulted in a reduction in the propeller power (AZIPOD’S + FIXPOD) demand by 0.1 ÷ 0, 4 MW, while maintaining practically the same ship speed. If taking into account the fact that the power consumption of the activated ALS blowers was 0.34 ÷ 0.63 MW, it appears that the decrease of propeller power demand is balanced by the increase of power consumption by the operated blowers. Practically the fuel consumption of a ship does not change substantially. It can therefore be concluded that the operation of ALS did not improve the ship’s propulsion efficiency. This is confirmed by the power curve shown in Figure 11. Contrary to the propeller curve for the chemical tanker MT Amalienborg (Figure 7), switching the ALS ON did not shift curve towards the “light propeller”, the propulsion system operating points for a passenger vessel with diesel-electric propulsion were dispersed (Figure 11) and did not clearly divide the propulsion curve with ALS ON (lighter propeller) and OFF (heavier propeller).

## 6. Conclusions

Based on the conducted analyses of the available literature and records of operating data, it can be stated that:

- the benefits of ALS use seem doubtful (only at the ship design stage, the application of this system improves the EEDI value, which is interesting for ship designers and ship-owners).
- the use of the ALS for the entire ship’s speed range is not beneficial, there are minimum and maximum speeds beyond which the use of the system does not give the assumed savings.
- equipment included in the structure of the ALS, including main blowers, require high investment outlays and high operating costs.
- maintaining the same size and evenly distributed air bubbles under the hull surface is a difficult task. Changing the diameter of the air bubbles significantly affects their distribution under the hull and may significantly reduce the effect of reducing the ship’s drag. Although features such as protruding ridges on the edges of the hull can help maintain the air layer, but these elements contribute to increased drag and stability of the ship, especially in heavy seas.
- it is difficult to counteract the effect of air bubbles being sucked in by the propeller, causing noise and vibration and leading to a reduction of the propeller efficiency [31].

The authors’ observations included in this article, which consist in a rather critical approach to the use of the ALS on ships, are proved in reality. It seems that in recent years, the interest in using this system on newly designed and built units has decreased, and if it appears, it is usually dictated by the need to obtain the recommended EEDI value.

## Figures and Tables

**Table 1:** The list of cruise vessels with Air Lubrication System delivered 2015–2019.

Year	Vessel Name	Type	System
2015	Quantum of the Sea	Cruise	Foreship
2016	AIDAprima	Cruise	MALS
2017	AIDAperla	Cruise	MALS
2017	Norwegian Joy	Cruise	Silverstream
2018	Diamond Princess	Cruise	Silverstream

**Table 2:** Calculated parameters of propulsion system, based on the operating parameters recorded during passenger vessel sailing with ON and OFF ALS.

Probe No.	ALS State	ALS Power Consumption	^1^ Propeller Power Distribution	^2^ Fuel Consumption Difference	Fuel Consumption Difference	^3^ Propeller Power Difference	^4^ Summary Propulsion Power Difference	^5^ Average Speed Difference
-	-	[MW]	[%]	[kg/h]	[%]	[MW]	[MW]	[kn]
-	-	A	B	C	D	E	F	G
6	off		69%					
7	on	0.47	68%	97	0,8	0.3	−0.17	0.3
9	on	0.51	66%				−0.31	
10	off		69%	−107	-0.9	−0.3	−0.30	0.1
16	off		71%				−1.30	
17	on	0.47	70%	125	1.0	0,7	0.23	0.1
19	on	0.51	69%				−0.51	
20	off		70%	−68	-0.6	−0.1	−0.10	−0.2
22	off		71%	0		1.0	1.00	
23	on	0.51	68%	75	0,6	0.5	−0.01	−0.1
24	on	0,51	69%				−0.71	
25	off		69%	299	2.4	−0.6	−0.60	0.0
26	off		78%				−2.10	
27	on	0.35	77%	237	1.7	−0.3	−0.65	−0.4
28	off		72%				−0.10	
29	on	0.48	69%	20	0.2	0.8	0.32	−0.4
30	on		71%	−101	−0.8	−0.2	−0.20	0.0
31	on	0.63	69%	146	1.2	0.6	−0.03	−0.1
32	off		68%	−95	−0.8	0.3	0.30	−0.1
33	off		71%				−0.60	
34	on	0.46	69%	189	1.5	0.0	−0.46	0.0
37	on	0.46	68%				−0.36	
38	off		70%	−102	−0.8	−0.3	−0.30	−0.1

^1^ Propeller Power distribution-the part of total power produced on the vessel used for propulsion purposes. ^2^ Fuel consumption difference-difference of fuel consumption when ALS is ON or OFF. ^3^ Propeller Power difference-difference of propeller power consumption before and after start ALS. ^4^ Summary Propulsion Power difference-difference of propulsion purpose power consumption before and after start ALS. ^5^ Average speed difference-difference of ship speed before and after start ALS.

## Data Availability

Not applicable.

## Abbreviations

The following abbreviations are used in this manuscript:

NOx	Nitrogen Oxides
SOx	Sulphur Oxides
CO	Carbon Oxides
HC	Hydrocarbons
PM	Particular Matter
IMO	International Maritime Organization
MARPOL	MARine POLution convention
EEDI	Energy Efficiency Design Index
ALS	Air Lubrication System
R	Total Resistance
R_P_	Pressure Resistance
R_W_	Wave Resistance
R_F_	Frictional Resistance
R_A_	Aerodynamic Resistance
R_H_	Hydrodynamic Resistance
R_VP_	Viscous Pressure Resistance
c	Dimensionless Drag Coefficient
ρ	Fluid Density
ν	Velocity
S	Surface of the ship’s hull
AL	Air Lubrication
MALS	Mitsubishi Air Lubrication System
WAIP	Winged Air Induction Pipe System-R&D Engineering
SAVER Air	SAVER System-Samsung Heavy Industries
BHP	Brake Horse Power
GPS	Global Positioning System
MT	Motor Tanker
kn	Knots
MBB	Main Bus Bars
AZIPOD	Azimuthal stern thruster
FIXPOD	Fixed gondola thruster
BT	Bow Thruster
PP	Propeller Power
ALSP	Air Lubricating system Power
SPP	Summary Propulsion Power
